# High Genetic Diversity and Insignificant Interspecific Differentiation in *Opisthopappus* Shih, an Endangered Cliff Genus Endemic to the Taihang Mountains of China

**DOI:** 10.1155/2013/275753

**Published:** 2013-12-17

**Authors:** Rongmin Guo, Lihua Zhou, Hongbo Zhao, Fadi Chen

**Affiliations:** ^1^Department of Ornamental Horticulture, School of Landscape Architecture, Zhejiang Agriculture and Forestry University, Lin'an, Hangzhou, Zhejiang 311300, China; ^2^Nurturing Station for State Key Laboratory of Subtropical Silviculture, Lin'an, Hangzhou 311300, China; ^3^College of Horticulture, Nanjing Agricultural University, Nanjing 210095, China

## Abstract

*Opisthopappus* Shih is endemic to the Taihang Mountains, China. It grows in the crevice of cliffs and is in fragmented distribution. This genus consists of two species, namely, *O. taihangensis* (Ling) Shih and *O. longilobus* Shih, which are both endangered plants in China. This study adopted intersimple sequence repeat markers (ISSR) to analyze the genetic diversity and genetic structure from different levels (genus, species, and population) in this genus. A total of 253 loci were obtained from 27 primers, 230 of which were polymorphic loci with a proportion of polymorphic bands (*PPB*) of up to 90.91% at genus level. At species level, both *O. taihangensis* (*PPB* = 90.12%, *H* = 0.1842, and *I* = 0.289) and *O. longilobus* (*PPB* = 95.21%, *H* = 0.2226, and *I* = 0.3542) have high genetic diversity. Their respective genetic variation mostly existed within the population. And genetic variation in *O. longilobus* (84.95%) was higher than that in *O. taihangensis* (80.45%). A certain genetic differentiation among populations in *O. taihangensis* was found (*G*_*st*_ = 0.2740, Φ_*st*_ = 0.196) and genetic differentiation in *O. longilobus* was very small (*G*_*st*_ = 0.1034, Φ_*st*_ = 0.151). Gene flow in different degrees (*N*_*m*_ = 1.325 and 4.336, resp.) and mating system can form the existing genetic structures of these two species. Furthermore, genetic differentiation coefficient (*G*_*st*_ = 0.0453) between species and the clustering result based on the genetic distance showed that interspecific differentiation between *O. taihangensis* and *O. longilobus* was not significant and could occur lately.

## 1. Introduction

*Opisthopappus* Shih (Asteraceae) is a genus of perennial herbaceous plants with two species, namely, *O. taihangensis* (Ling) Shih and *O. longilobus* Shih [1]. These two species are endemic to the Taihang Mountains (China) on the borders between Shanxi, Hebei, and Henan provinces. They grow mostly on cliff cracks, rock gaps in open forests below cliffs, and infertile soil at an elevation of 1000 m [1]. The flowers are white and large, with remarkably high ornamental and medicinal values [2] and potential genetic resource for chrysanthemum improvement [3–5]. Given the changes in their habitats and manmade damage, their distribution range is decreasing continuously. At present, these species are in an endangered status, listed as national rare and endangered plants in China [6]. *O. taihangensis* and *O. longilobus* can be distinguished according to their leaf morphology and the presence or absence of bracts below involucres [1]. The former is distributed in the south of the Taihang Mountain, mainly in Henan and Shanxi; meanwhile, the latter is distributed in the north of the Taihang Mountains, mainly in Hebei. The distribution of these two species basically regards the border between Hebei and Henan (36.07°N latitude) as the boundary. Based on relative specimen records, an overlapping distribution of these two species exists in the Linzhou district of the north of Henan.

Daxinganling Mountains—Taihang Mountains—Wu Mountains—Xuefeng Mountains is the borderline of the second and third classes of China Relief. The Taihang Mountains is northeast-southwest trend mainly but some parts nearly south-north trend, with the terrain of high north and low south. The altitude of most parts is above 1,200 m. The mountain is precipitous in the east and gradual in the west. Shanxi plateau is connected to the west, and the east part is from middle mountains, low mountains, hills to plains. This terrain allows the growth of rich seed plants in this area. The geographic distribution type of genera in seed plants is diversiform with a great amount of ancient, relict, original and endemic taxa of China, in which *Opisthopappus* and *Taihangia *Yu et Li are endemic taxa in this area [1, 7–9].

Generally, species with small geographic ranges tend to maintain less genetic diversity than those that are geographically widespread [10]. Several plant species distributed in cliffs show a relatively low genetic diversity [11–14], whereas a few of which have high genetic diversity [12, 15, 16]. High genetic diversity maintained in rare plants is attributable to many factors [17], such as the recent reduction of population size plus insufficient time for isolation, extensive and recurrent gene flow, outcrossing, and fruit dispersal via wind [18–20]. As an ancient habitat, cliffs provide a refuge for plants distributed therein to escape animal feed, interspecies competition, and human disturbance. However, a great heterogeneity also exists on a small scale; for example, differences are found in the proportion of plants to cliff cracks, soil quantity and quality, rock type, weathering degree, and number of surrounding plants [15]. Meanwhile, the differences in the orientation of vertical cliffs also cause differences in illumination, temperature, relative humidity, soil moisture, plant density, and wind speed [21]. This type of local environment diversity causes plants to exhibit polymorphism, which eventually results in high genetic diversity in populations [15, 16]. In *Lloydia serotina*, species in cliffs have similar or higher genetic diversity than those in other areas [12]. *Taihangia rupestris* Yu et Li [22, 23] also shows high genetic diversity. The results of cluster analysis show that *T. rupestris* var. *rupestris* and *T. rupestris* var. *ciliate* have significant genetic differentiation.

Currently, studies on *Opisthopappus* mainly focus on tissue culture [24, 25], karyokinesis [26], meiosis [27, 28], intergeneric crossing [3, 5], pollen morphology [29], and phylogenetic evolution [4]. Wang and Yan [30] reported population genetic structure of two species in *Opisthopappus* by sequence related amplified polymorphism markers (SRAP). The results showed high genetic diversity existed in populations of the two species. The main aims of this study is to evaluate the genetic diversity and population genetic structure from different levels (genera, species, and populations) in *Opisthopappus* by adopting the inter-simple sequence repeat (ISSR) molecular marker. Furthermore, according genetic variation and differentiation, interspecific relationship and differentiation were analyzed and realized. The results can provide scientific data and theoretical foundation for the effective protection, sustainable development, and utilization of *Opisthopappus *species.

## 2. Materials and Methods

**Figure 1:**
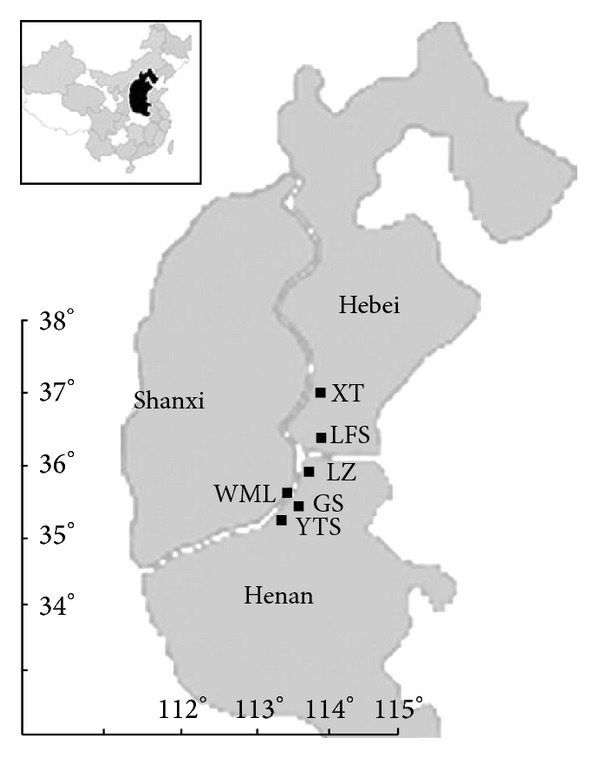
Geographic distribution and sampling sites in *Opisthopappus.*

*Study Species.* Six natural populations consisting of three *O. taihangensis* (i.e., YTS, GS, and WML) and three* O. longilobus* (LZ, LFS, and XT) populations were collected from the Taihang Mountains in Henan, Shanxi, and Hebei province to serve as materials (Figure 1). A total of 200 individuals were collected (Table 1). The spacing distance between individuals was more than 3 m. Fresh leaves in robust growth were selected for rapid drying using anhydrous silica gel. After drying completely, the leaves were placed in an ultra-cold storage freezer for cold storage. 

*Extraction of Genome DNA and ISSR-PCR Amplification.* An ample amount of dried leaves was taken to extract genomic DNA using the improved CTAB method [31]. The mass and content of DNA were measured using a spectrophotometer to dilute it to 20 ng/*μ*L. Twenty-seven primers with high polymorphism and good repeatability were screened from 54 ISSR primers for PCR amplification. The total volume of the ISSR-PCR reaction system was 20 *μ*L, including 20 ng to 50 ng template DNA, 1.5 mmol/L Mg^2+^, 1 × *Taq *DNA polymerase buffer solution (containing 10 mmol/L Tris-Hcl, 50 mmol/L Kcl, pH 9.0), 1.0 *μ*mol/L primer, 0.25 mmol/L dNTPS, and 1.0 U *Taq *DNA polymerase. The amplification program used was as follows: predenaturation for 5 min at 94°C; denaturation for 45 s at 94°C; annealing for 40 s at 55°C; extension for 70 s at 72°C, 37 cycles; and a final extension for 7 min at 72°C. 

*Genetic Diversity Analysis.* According to the bands in the electrophoretogram, the positions with the same migration rate on the gel and with DNA bands were recorded as “1”, and those without DNA bands were recorded as “0.” POPGEN 1.32 software [32] was used to calculate the genetic parameters: (1) percentage of polymorphic loci (*PPB*); (2) number of alleles (*N*_*a*_) and number of effective alleles (*N*_*e*_); (3) expected heterozygosity (*He*); (4) Nei's genetic diversity index (*H*) and Shannon's information index (*I*); (5) total genetic diversity index (*H*_*t*_) and genetic diversity index in populations (*H*_*s*_); (6) genetic differentiation coefficient (*G*_*st*_ = 1 − *H*_*s*_/*H*_*t*_) and gene flow [*N*_*m*_ = (1 − *G*_*st*_)/2*G*_*st*_]; and (7) Nei's genetic distance (*D*) and genetic identity (*I*_*N*_). Analysis of molecular variance (AMOVA) in GENALEX 6.4 software package [33] was used to calculate the genetic variation and genetic distance (Φ_*st*_). Based on Nei's genetic distance, NTSYS-pc 2.10e software [34] was used to conduct the unweighted pair-group method with arithmetic mean (UPGMA) cluster analysis. In addition, TFPGA software [35] was adopted to perform the Mantel test of correlation between the genetic and geographical distance between species groups.

## 3. Results

*Genetic Diversity.* A total of 253 ISSR bands were amplified, 230 of which were polymorphism bands. The *PPB* at genus level was 90.91%, 90.12% for *O. taihangensis,* and 95.21% for *O. longilobus*. Generally, at genus level, *Opisthopappus* showed higher genetic diversity. Nei's total genetic diversity index of *Opisthopappus* (*H*_*t*_) was 0.2139 (Table 2). Meanwhile, at species level, the total genetic diversity index (*H*_*t*_ = 0.2173) and genetic diversity index in populations (*H*_*s*_ = 0.1948) of *O. longilobus* Shih were slightly higher than those of *Opisthopappus* Shih (*H*_*t*_ = 0.1930, *H*_*s*_ = 0.1401).

The percentage of polymorphic loci (*PPB*), number of alleles (*N*_*a*_), number of effective alleles (*N*_*e*_), expected heterozygosity (*He*), Nei's genetic diversity index (*H*), and Shannon's information index (*I*) at genus level were 90.91%, 1.9467, 1.3452, 0.1675, 0.2121, and 0.3372, respectively (Table 3). At species level, all genetic parameters of *O. longilobus* were higher than those of *O. taihangensis*. Moreover, among the three populations of *O. taihangensis*, all genetic parameters of the GS population were the highest, whereas those of WML were the lowest. Meanwhile, among three populations of *O. longilobus*, all genetic parameters of the LZ population were the highest, whereas those of LFS were the lowest. At population level, all the genetic parameters of LZ were the highest, whereas those of WML were the lowest (Table 3).

*Genetic Differentiation and Gene Flow.* Genetic differentiation coefficient (*G*_*st*_) between two species of *Opisthopappus* was 0.0453, which was lower than that among populations at species level (Table 2). Furthermore, genetic differentiation among populations in *O. taihangensis* was slightly higher than *O. longilobus *(Table 2).

At population level, genetic differentiation coefficient (Φ_*st*_) arranged from 0.122 to 0.427 (i.e., an average of 0.240) (Table 4). The coefficient between LFS and WML was the highest (0.427), whereas that between XT and LFS was the lowest (0.122). For *O. taihangensis*, genetic differentiation between GS and YTS, GS and WML, and YTS and WML increased gradually; similarly, for *O. longilobus*, genetic differentiation between XT and LFS, LZ and XT, and LZ and LFS also increased gradually (Table 4).

*Genetic Variation and Structure. *The AMOVA results showed that most of genetic variation in *Opisthopappus* existed within population (77.87%), following among populations (16.15%), and between species (5.98%) (Table 5). Most of the genetic variation of the two species, respectively, also existed within populations. Moreover, genetic variation of *O. longilobus* within populations (84.95%) was slightly higher than that of *O. taihangensis* (80.45%).

**Figure 2:**
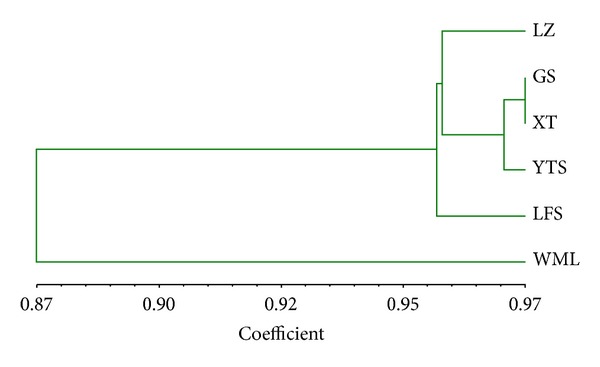
UPGMA cluster analysis of the six populations (two species) based on ISSR analysis.

*Cluster Analysis and Correlation between Geographical Distance and Genetic Distance. *At population level, genetic distance between XT and GS population was lowest (0.0277) and that between LZ and WML was highest (0.1552) (Table 6). According to UPGMA cluster analysis based on Nei's genetic distance (Figure 2), the respective populations of two species were not clustered into one clade at first, while XT and GS populations were clustered firstly and were then composed of YTS and LZ to a clade. WML population had the largest distance to all other populations; thus, it located in the basal of the clustering tree.

The results of Mantel tests showed that geographical distance among populations had no significant correlation with genetic distance (*r* = −0.2419, *P* = 0.1845). Furthermore, at species level, there was no significant correlation between geographical distance and genetic distance in *O. taihangensis* (*r* = 0.7154, *P* = 0.3380) and *O. longilobus *(*r* = 0.6011, *P* = 0.3510), respectively.

## 4. Discussion

*Genetic Diversity.* In *Opisthopappus*, not only at genus level but also at species level, there was high genetic diversity. Nei's genetic diversity and Shannon's information index of *O. taihangensis* (Ling) Shih and *O. longilobus* Shih were higher than those of herbaceous plants (*H* = 0.162, *I* = 0.238) and plants in narrow fields or endangered plants (*H* = 0.157, *I* = 0.249) [36, 37] but were slightly lower than those of *Taihangia rupestris*, another cliff species endemic to the same area [22, 23]. These indicated that two species of *Opisthopappus* both had higher genetic diversity. The degree of genetic diversity in *O. longilobus* was slightly higher than that of *O. taihangensis*. The AMOVA analysis showed that most of the genetic variation existed within populations and LZ had the highest genetic diversity among six populations. *T. rupestris* also had similar genetic diversity and genetic structure [22, 23].

**Figure 3:**
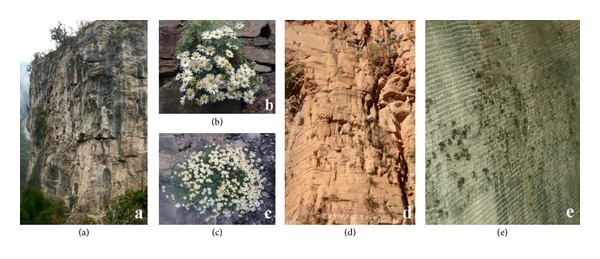
Cliff habitats and flowering plants in *Opisthopappus*. (a and b) *O. taihangensis*; (c and d) *O. longilobus*; (e) distributing condition of *O. taihangensis* in a manmade reservoir dam (more and more plants from the upper to the lower of dam and distribution scope showing triangle) and that reflecting the seed dispersal by wind and gravity.

These two taxa (i.e., *Opisthopappus *and *Taihangia*) endemic to the Taihang Mountains showed similar genetic structures. For *T. rupestris*, ancient origin and comprehensive reproductive mechanisms might be the key contributing factors for its higher genetic diversity [23]. *T. rupestris* produced rich genetic variation through sexual reproduction during the long-term evolutionary process. The coexistence of asexual and sexual reproduction caused these variations to accumulate and spread [23]. Meanwhile, higher genetic diversity in *Opisthopappus* was closely related to its special habitats, population regeneration, and dispersal. Individuals of *Opisthopappus* all grow in cliff cracks with a slope of nearly 90° (Figures 3(a)–3(d)). No conditions for vegetative propagation are present, given that its seeds are small and light and easily spread by gravity and wind (Figure 3(e)). It can be inferred that sexual reproduction is the main reproductive mode under natural conditions. This reproductive mode is beneficial to gene flow among individuals and among populations, resulting in an increase and maintenance of the degree of genetic diversity. Moreover, *O. longilobus* is distributed in the north of the Taihang Mountains. The environmental conditions such as precipitation, humidity, and temperature therein are more wretched than those in the distribution area of *O. taihangensis*. Therefore, natural regeneration can hardly occur, with slower alternation of generations. Hence, its measured genetic diversity is higher than that of *O. taihangensis*. LZ population has the highest genetic diversity, which may be attributable to the fact that it is in the intermediate zone between *O. taihangensis* and *O. longilobus* with special habitats, resulting in rich variation within population and an increased degree of genetic diversity.

*Genetic Differentiation and Interspecific Differentiation.* Genetic differentiation coefficient of *O. taihangensis* (*G*_*st*_) is higher than that of endemic and perennial plants (*G*_*st*_ = 0.18 and 0.19, resp.) [37, 38]. Furthermore, genetic differentiation coefficient of *O. longilobus* Shih is lower than that of endemic and perennial plants. These indicate the presence of a certain genetic differentiation among populations in *O. taihangensis*; however genetic differentiation of *O. longilobus* is relatively small. Gene flow at different degrees (1.325 in *O. taihangensis* and 4.336 in *O. longilobus*) and mating system (sexual reproduction) can be the contributing factors to the current genetic structures in this genus. Low gene flow may cause population to adapt to the local ecological environment, thus accelerating genetic differentiation [39]. Wright [40] asserted that when gene flow *N*_*m*_ > 1, genetic differentiation among populations due to genetic drift can be prevented. For *O. taihangensis* or *O. longilobus*, AMOVA showed that genetic variation mostly existed within population (80.45% and 84.95%, resp.). A similar phenomenon was found in *T. rupestris* [22, 23], implying that habitat fragmentation and particularity do not cause significant genetic differentiation among populations in these endangered cliff plants endemic to the Taihang Mountains, and instead, promote them to adapt to the special habitats to sustain higher genetic diversity.

A significantly positive correlation exists between genetic and geographical distance for *T. rupestris *[22, 23] and *Centaurea corymbosa* Pourret [41], another rare, cliff-dwelling species. By contrast, in the studies on *Opisthopappus*, the Mantel test revealed that no significant correlation exists between genetic and geographical distance (*r* = −0.2419, *P* = 0.1845). Geographical isolation cannot explain the population differentiation that occurred in *Antirrhinum charidemi* and *A. valentinum *(endemic species distributed narrowly) [42] and *Arctomecon humilis* (an endangered plant) [43]. The absence of correlation between geographical and genetic distance in these species may be related to the rude interference of human beings at the least [43]. Human activity may break the balance between population migration and adaption, deepen habitat fragmentation [42], reduce the size of proper migration sites, and increase the isolated distance among populations [22, 44, 45]. *O. taihangensis* and *O. longilobus* both have a certain medical value, which led to serious human picking and mining. These activities cause great impact on the effective size of populations and their dispersal, affecting the natural gene flow among populations. They can explain the absence of correlation between geographical and genetic distance.

In *T. rupestris*, the clustering of the different populations into the two major clades agreed with the geographical distribution and its classification into the two varieties [22, 23]. The moderate degree of gene flow shaped the genetic profiles of *T. rupestris*, homogenizing the genetic diversity of the two varieties, namely, *T. rupestris* var. *rupestris* and *T. rupestris* var. *ciliate *[23]. The two varieties were classified based on the difference in their leaf morphology [7]. They are distributed in the south and north of the Taihang Mountains, respectively. *O. taihangensis* and *O. longilobus* are similar to *T. rupestris* in terms of morphological difference and geographic distribution; however, the clustering result of this study shows that the respective populations of these two species do not gather but cross-cluster. Hence, interspecific genetic differentiation between two species is not significant. Their interspecific differentiation is significantly later than the two varieties of *T. rupestris* [1]. *O. taihangensis* and *O. longilobus* are classified according to the split series of the upper leaves and the presence or absence of bracts below involucres. In *O. taihangensis*, the leaves are bipinnatifid and have pubescences attached to both sides; it does not have bracts below involucres. By contrast, most of the leaves of *O. longilobus* are pinnatifid except for basal leaves (bipinnatifid or near the bipinnatifid) and all leaves are smooth without pubescences; in addition, it has a pair of bracts below involucres [1]. Almost no genetic differentiation was found between the two subspecies of *Dryas octopetala* L. (Rosaceae) despite their significant morphological differences [46]. For *Opisthopappus*, the speed of interspecific genetic differentiation can be less than the speed of morphological differentiation, which may be closely related to mating system. Sexual reproduction will promote gene flow among populations, even between species, reducing the interspecific genetic differentiation. Meanwhile, in *T. rupestris*, the main mode of population regeneration is vegetative propagation [22], which is favorable to fix genetic characteristics in the level of population, subspecies, or species and thus enhance genetic differentiation.

*Genetic Diversity and Resource Protection. *The degree of genetic diversity to a certain extent reflects the capacity of species to adapt to the external environment. It not only restricts the capacity of individual and species to adapt to the environmental changes but also provides very important information for the realization and protection of species [14, 47]. The results in this study have shown that the endangered status in *Opisthopappus* is not caused by the degree of genetic diversity but by population regeneration block and the difficulty to recovery due to habitat destruction and fragmentation. Therefore, an effective protection procedure should be developed to avoid a serious endangered situation; for example, in situ conservation through establishing a nature reserve, ex situ conservation in areas with serious habitat destruction, and manual promotion to natural regeneration can be carried out.

## Figures and Tables

**Table 1:** Locations of six natural populations in *Opisthopappus *Shih.

Populations	Locality	Sample size	Longitude (*E*)	Latitude (*N*)	Altitude (*m*)
*O. taihangensis *					
YTS	Yuntaishan Mountains, Xiuwu, Henan province	49	113.138°	35.450°	1070
GS	Guanshan Mountains, Huixian, Henan province	49	113.530°	35.562°	1057
WML	Wangmangling Mountains, Lingchuan, Shanxi province	8	113.594°	35.687°	1550
*O. longilobus *					
LZ	Taihang Grand Canyon, Linzhou, Henan province	42	113.691°	36.170°	684
LFS	Lufengshan Mountains, Fengfeng, Hebei province	18	113.918°	36.404°	999
XT	Xingtai Grand Canyon, Xingtai, Hebei province	34	113.872°	37.114°	532

**Table 2:** Nei's total genetic diversity index (*H*_*t*_), genetic diversity index in populations (*H*_*s*_), genetic differentiation coefficient (*G*_*st*_), and gene flow (*N*_*m*_) in *Opisthopappus*.

Taxa	*H*_*t*_	*H*_*s*_	*G*_*st*_	*N*_*m*_
*Opisthopappus *	0.2139 ± 0.0316	0.2042 ± 0.0286	0.0453	10.562
*O. taihangensis *	0.1930 ± 0.0314	0.1401 ± 0.0149	0.2740	1.325
*O. longilobus *	0.2173 ± 0.0301	0.1948 ± 0.0235	0.1034	4.336

**Table 3:** Percentage of polymorphic loci (*PPB*), number of alleles (*N*_*a*_), number of effective alleles (*N*_*e*_), expected heterozygosity (*He*), Nei's genetic diversity (*H*), and Shannon's information index (*I*) in *Opisthopappus. *

Populations	*PPB*	*N*_*a*_	*N*_*e*_	*He*	*H*	*I*
*O. taihangensis *						
YTS	62.45%	1.6245 ± 0.4852	1.2748 ± 0.3729	0.1588 ± 0.0124	0.1588 ± 0.1966	0.2423 ± 0.2755
GS	71.54%	1.7154 ± 0.4521	1.2713 ± 0.3375	0.1663 ± 0.0114	0.1663 ± 0.1806	0.2618 ± 0.2547
WML	38.74%	1.3874 ± 0.4881	1.1482 ± 0.2637	0.0954 ± 0.0094	0.0954 ± 0.1493	0.1538 ± 0.2220
Mean	57.58%	1.5758 ± 0.4751	1.2314 ± 0.3247	0.1402 ± 0.0111	0.1402 ± 0.1755	0.2193 ± 0.2507
Species level	90.12%	1.9012 ± 0.2990	1.3062 ± 0.3569	0.1401 ± 0.0065	0.1842 ± 0.1867	0.2895 ± 0.2562
*O. longilobus *						
LZ	93.28%	1.9328 ± 0.2509	1.3439 ± 0.3286	0.2157 ± 0.0105	0.2157 ± 0.1675	0.3443 ± 0.2242
LFS	54.15%	1.5415 ± 0.4993	1.2796 ± 0.3532	0.1665 ± 0.0120	0.1665 ± 0.1913	0.2538 ± 0.2741
XT	81.42%	1.8142 ± 0.3897	1.3345 ± 0.3561	0.2021 ± 0.0116	0.2021 ± 0.1851	0.3155 ± 0.2547
Mean	76.28%	1.7628 ± 0.3800	1.3193 ± 0.3460	0.1948 ± 0.0114	0.1948 ± 0.1813	0.3045 ± 0.2510
Species level	95.21%	1.9921 ± 0.0887	1.3599 ± 0.3403	0.1948 ± 0.0066	0.2226 ± 0.1710	0.3542 ± 0.2245
*Opisthopappus *						
Mean	66.93%	1.6693 ± 0.4276	1.2754 ± 0.3353	0.1675 ± 0.0113	0.1675 ± 0.1784	0.2619 ± 0.2509
Genus level	90.91%	1.9467 ± 0.1939	1.3452 ± 0.3450	0.1675 ± 0.0047	0.2121 ± 0.1764	0.3372 ± 0.2340

**Table 4:** Genetic differentiation of PhiPT analysis between populations.

Populations	YTS	GS	WML	LZ	LFS	XT
YTS	∗∗∗∗					
GS	0.131	∗∗∗∗				
WML	0.361	0.328	∗∗∗∗			
LZ	0.239	0.219	0.309	∗∗∗∗		
LFS	0.274	0.251	0.427	0.180	∗∗∗∗	
XT	0.156	0.144	0.311	0.142	0.122	∗∗∗∗

**Table 5:** The analysis of molecular variance (AMOVA) in *Opisthopappus. *

Source of variation	Degrees of freedom	Sums of squares	Variance component	Variation (%)	P*
*Opisthopappus *					
Between species	1	423.0082	1.921	5.98	<0.001
Among populations	4	719.6363	5.182	16.15	<0.001
Within population	194	4847.7555	24.988	77.87	<0.001
Total	**199**	**5990.4000**	**32.091**		
*O. taihangensis *					
Among populations	2	354.9223	5.195	19.55	<0.001
Within population	103	2201.3418	21.372	80.45	<0.001
Total	**105**	**2556.2641**	**26.567**		
*O. longilobus *					
Among populations	2	364.7140	5.153	15.05	<0.001
Within population	91	2646.4136	29.081	84.95	<0.001
Total	**93**	**3011.1276**	**34.234**		

*P values are the probabilities of having a more extreme variance component than the observed values by chance alone. Probabilities calculated by 1000 random permutations of individuals across populations.

**Table 6:** Nei's genetic identity (above diagonal) and genetic distance (below diagonal) in populations.

Populations	YTS	GS	WML	LZ	LFS	XT
YTS	∗∗∗∗	0.9681	0.8642	0.9484	0.9410	0.9684
GS	0.0324	∗∗∗∗	0.9044	0.9549	0.9531	0.9727
WML	0.1460	0.1004	∗∗∗∗	0.8562	0.8648	0.8688
LZ	0.0529	0.0462	0.1552	∗∗∗∗	0.9518	0.9634
LFS	0.0609	0.0480	0.1453	0.0494	∗∗∗∗	0.9722
XT	0.0321	0.0277	0.1407	0.0372	0.0282	∗∗∗∗
